# Mechanisms of *Actinidia chinensis* Planch in treating colon cancer based on the integration of network pharmacology, molecular docking, and experimental verification

**DOI:** 10.1186/s41065-023-00303-x

**Published:** 2023-12-15

**Authors:** Jin-Fang Chen, Shi-Wei Wu, Zi-Man Shi, Yan-Jie Qu, Min-Rui Ding, Bing Hu

**Affiliations:** 1 Institute of Traditional Chinese Medicine in Oncology, Longhua Hospital, Shanghai University of Traditional Chinese Medicine, Shanghai, 200032 People’s Republic of China; 2 Department of Oncology, Longhua Hospital, Shanghai University of Traditional Chinese Medicine, Shanghai, 200032 People’s Republic of China; 3 Department of Neurology, Longhua Hospital, Shanghai University of Traditional Chinese Medicine, Shanghai, 200032 People’s Republic of China; 4 Department of Traditional Chinese Medicine, Ruijin Hospital, Shanghai Jiao Tong University School of Medicine, Shanghai, 200025 People’s Republic of China

**Keywords:** *Actinidia chinensis* Planch, Colon cancer, Network pharmacology, Molecular docking, Apoptosis, PI3K-Akt signaling pathway

## Abstract

**Background:**

As an anticancer Chinese herbal medicine, the effective components and mechanism of *Actinidia chinensis* Planch (ACP, Tengligen) in the treatment of colon cancer are still unclear. In the present study, the integration of network pharmacology, molecular docking, and cell experiments was employed to study the effective mechanism of ACP against colon cancer.

**Methods:**

The Venn diagram and STRING database were used to construct the protein–protein interaction network (PPI) of ACP-colon cancer, and further topological analysis was used to obtain the key target genes of ACP in colon cancer. The Gene Ontology (GO) and Kyoto Encyclopedia of Genes and Genomes (KEGG) enrichment analyses were used to visualize the related functions and pathways. Molecular docking between key targets and compounds was determined using software such as AutoDockTools. Finally, the effect of ACP on CT26 cells was observed in vitro.

**Results:**

The study identified 40 ACP-colon key targets, including CASP3, CDK2, GSK3B, and PIK3R1. GO and KEGG enrichment analyses found that these genes were involved in 211 biological processes and 92 pathways, among which pathways in cancer, PI3K-Akt, p53, and cell cycle might be the main pathways of ACP against colon cancer. Molecular docking verified that the key components of ACP could stably bind to the corresponding targets. The experimental results showed that ACP could inhibit proliferation, induce apoptosis, and downregulate the phosphorylation of PIK3R1, Akt, and GSK3B in CT26 cells.

**Conclusion:**

ACP is an anti-colon cancer herb with multiple components, and involvement of multiple target genes and signaling pathways. ACP can significantly inhibit proliferation and induce apoptosis of colon cancer cells, which may be closely related to the regulation of PI3K/AKT/GSK3B signal transduction.

## Introduction

Colorectal cancer (CRC) is a common malignancy with the third and second highest incidence and mortality rates of 10.0% and 9.4% worldwide, respectively [1]. Surgery can effectively eradicate CRC, but even in combination with adjuvant chemotherapy and other treatments, approximately 45% of patients still develop recurrence and metastasis after surgery [2]. The combination of traditional Chinese medicine (TCM) with radiotherapy, chemotherapy, biological targeting, and immunotherapy has a good effect on the treatment of CRC, and it can improve the therapeutic effect, reduce toxic and side effects, ameliorate clinical symptoms, improve the quality of life, and prolong the survival time [3].

In recent years, TCM-based herbal medicines have gained more and more recognition worldwide and have been widely used in the field of anticancer, among which *Actinidia chinensis* Planch (ACP, Tengligen) is one of the representative anticancer herbal medicines that is able to play a therapeutic role in a variety of cancers. In both in vitro and in vivo experiments, Gao et al. [4] discovered that ACP increased the build-up of reactive oxygen species (ROS), down-regulated the expression of mesenchymal-related proteins like vimentin and N-cadherin, and also down-regulated the expression of anti-ferroptosis proteins like GPX4 and xCT in gastric cancer HGC-27 cells. It is suggested that ACP is effective in preventing gastric carcinogenesis by inducing apoptosis, promoting iron death, and inhibiting mesenchymal phenotypes. By downregulating the protein expression of p-Akt, p-GSK3B, and β-catenin, ACP blocked the Akt/GSK-3β signaling pathway in breast cancer cells, which in turn had anti-proliferation, anti-migration, and anti-invasion effects [5]. Furthermore, Zheng et al. [6] showed that ACP extract also has a certain inhibitory effect on hypopharyngeal carcinoma, which can block E2F transcription factor 1 (E2F1)-mediated MNX1 antisense RNA 1 (MNX1-AS1), which in turn can have anti-proliferation, invasion, metastasis, and pro-apoptosis effects. However, more research is needed to determine the effective components and mechanisms of ACP in the treatment of CRC.

**Fig. 1:**
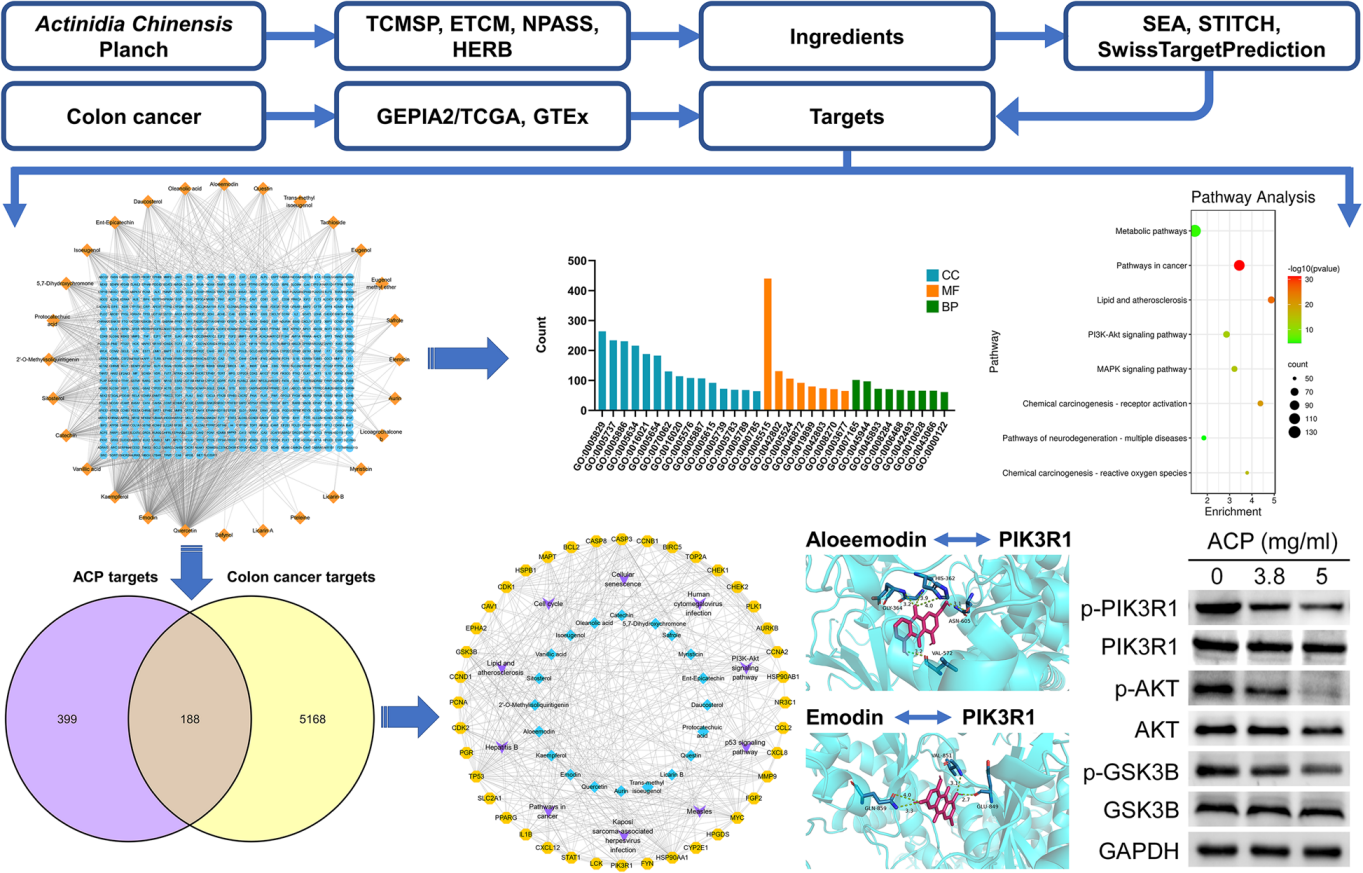
The whole framework of this study

The majority of recent research has focused on examining TCM's mechanism of action via a single target and pathway; its multi-target and multi-pathway functions have not been taken into account. In line with the holistic idea of TCM, network pharmacology is a novel research approach developed by Andrew L. Hopkins that has the potential to transform the single-target, single-method research paradigm into a systematic, comprehensive investigation of the relationships between drugs, targets, and diseases [7, 8]. Network pharmacology integrates information such as pharmacology, chemo-informatics, bioinformatics, and systems biology, which can predict the target and network effects of TCM compounds and clarify the relationship between “compounds-target-disease” to systematically reveal the effective mechanism of TCM [9]. In the present study, network pharmacology, molecular docking, and cytology experiments were used to study the effective active ingredients, related targets, and pathways of ACP for the treatment of colon cancer to provide a basis for the study and application of ACP. The flowchart for ACP in treating CRC is provided in Fig. 1.

## Materials and methods

### Extraction of the active compounds of ACP

The active compounds of ACP were collected from the Traditional Chinese Medicine Systems Pharmacology Database and Analysis Platform (TCMSP, http://tcmspw.com/tcmsp.php) [10], the Encyclopedia of Traditional Chinese Medicine (ETCM, http://www.tcmip.cn/ETCM/) [11], the Natural Product Activity and Species Source Database (NPASS, http://bidd.group/NPASS/index.php) [12], and the HERB (http://herb.ac.cn/) [13] databases. The information about the oral bioavailability (OB), drug-likeness (DL), or drug-likeness weight (DW) of these compounds was extracted from the TCMSP and ETCM databases and screened based on the criteria of OB ≥ 20%, DL ≥ 0.10, or DW ≥ 0.49.

### Prediction of compound-related targets

The SMILE numbers of the compounds were retrieved from the PubChem database (https://pubchem.ncbi.nlm.nih.gov/), and then submitted to the Similarity Ensemble Approach (SEA, http://sea.bkslab.org/), STITCH (http://stitch.embl.de/), and SwissTargetPrediction (http://swisstargetprediction.ch/) databases to predict targets based on structure similarity [14–16]. Converting gene names to official gene symbols in the Uniprot database (https://www.uniprot.org/). Max Tc ≥ 0.4, confidence score ≥ 0.4, and probability value ≥ 0.5 were used as screening criteria to predict the targets of ACP active ingredients.

### Identification of colon cancer-related targets

Colon cancer-related genes in the Cancer Genome Atlas (TCGA) and Genotype-Tissue Expression (GTEx) databases were identified using the Gene Expression Profiling Interactive Analysis (GEPIA2) online tool (http://gepia2.cancer-pku.cn/#index) with the criteria of *q* value < 0.01 and fold change (FC) ≥ 2 [17, 18].

### Network construction and topological analysis

The target genes of ACP against colon cancer were analyzed using the Venn online tool (https://www.omicshare.com/tools/Home/Soft/venn). The above target genes were then uploaded to the STRING database (https://string-db.org/) [19] to construct the PPI. The targets with a confidence score ≥ 0.7 were screened. Cytoscape 3.7.2 software [20] was used to establish the compound–target, compound–colon cancer overlapped target, compound–key target, and compound–target–pathway networks. In the compound–colon cancer overlapped target network, degree centrality (DC) ≥ 2 × median DC, betweenness centrality (BC) ≥ median BC, and closeness centrality (CC) ≥ median CC were used as criteria to screen the key targets of ACP.

### Gene ontology (GO) and pathway enrichment analysis

The ACP target genes were imported into the DAVID 6.8 database (https://david.ncifcrf.gov/) [21] for the enrichment analysis of the GO and Kyoto Encyclopedia of Genes and Genomes (KEGG) pathway [22–24]. GO and pathway enrichment were further visualized using online tools (http://www.bioinformatics.com.cn/, http://vip.sangerbox.com/home.html) [25].

### Molecular docking

The PDB format of protein structures was from the Protein Data Bank (PDB) (https://www.rcsb.org/) [26], and water molecules, small molecule ligands, and symmetry chains were removed in PyMOL v.3.8 software [27]. The protein file was further processed with operations such as hydrogenation, charge calculation, and addition of atomic types in AutoDockTools [28] and saved in PDBQT format. The SDF file of the valid compounds was retrieved from the Pubchem database, optimized in the Chem3D 15.1 module of ChemOffice software, and then converted to mol2 format as a small-molecule ligand. AutoDockTools was used to detect the root of the small molecule ligand and select the ligand rotatable bond, and the output was exported in PDBQT format. Molecular docking was performed after determining the docking region by using AutoDockTools to obtain the affinity score and the docked receptor and ligand. The binding sites were visualized using the PLIP website (https://projects.biotec.tu-dresden.de/plip-web/plip/) [29] and PyMOL v.3.8 software. The affinity score of ≤ -5 kJ/mol was used as a criterion for valid molecular binding.

## Materials

ACP granules (Lot No. 20050091, Quality Inspection Certificate No. XLS-09-MP-0013-0R01) were commercially produced by Sichuan Neo-green Pharmaceutical Technology Development Co., Ltd. (Chengdu, Sichuan, China). The product has a brownish-yellow granular texture and is soluble in water, so the aqueous extract of ACP granules was used for all subsequent experiments. RPMI-1640 medium, fetal bovine serum (FBS), penicillin–streptomycin, and trypsin were obtained from Gibco (Grand Island, NY, USA). The Cell Counting Kit-8 (CCK-8) assay, QuickBlock™ Blocking Buffer, and BCA Protein Assay Kit were purchased from Beyotime Biotechnology (Shanghai, China). FITC Annexin V apoptosis detection kit I was produced by BD Pharmingen™ (SanDiego, CA, USA). Antibodies against GAPDH, PIK3R1, p-PIK3R1, Akt, p-Akt, GSK3B, and p-GSK3B were purchased from Bioworld (St. Louis Park, MN, USA).

### Cell culture

Mouse colon cancer CT26 cells were obtained from The Cell Bank of Type Culture Collection of Chinese Academy of Sciences (Shanghai, China). CT26 cells were cultured in RPMI-1640 medium containing 10% FBS and 1% penicillin–streptomycin and placed in a 37 °C incubator containing 5% carbon dioxide, and logarithmic growth phase cells in good condition were taken for subsequent experiments.

### Cell viability assay

CT26 cells were inoculated at 2 × 10^3^/well in 96-well plates for 24 h, and then the cells were treated with different doses of ACP extracts (0, 200, 400, 800, 1600, 3200, 6400, and 12,800 μg/ml). The CCK-8 kit was added after 48 h of drug action to make it 10% in each well and incubated at 37 °C, protected from light, for 2 h. The absorbance of each well at 450 nm was detected using a microplate reader. Cell survival (%) = (experimental OD value/control OD value) × 100%.

### Cell apoptosis detected by flow cytometry

Logarithmic growth phase CT26 cells were taken and inoculated in 6-well plates at 1 × 10^5^/well. 24 h later, different concentrations of ACP extracts (0, 3.8, and 5 mg/ml) were added and treated. After 48 h of drug action, the cells were collected, washed twice with cold PBS, and resuspended with binding buffer. Then, 5 μl each of Annexin V-FITC and PI were added sequentially and stained for 15 min at room temperature, and apoptosis was analyzed by flow cytometry.

### Western blot

Protein expression and phosphorylation were evaluated by western blot [30]. CT26 cells treated as described above were collected, washed with PBS, and centrifuged, then lysed by adding RIPA buffer. The cell lysates were collected and then used for protein quantification with the BCA kit. Total proteins were separated by a 10% SDS-PAGE gel and then transferred to the PVDF membrane. The current size and time duration were determined depending on the molecular weight. The membrane was blocked with QuickBlock™ Blocking Buffer for 15 min and then incubated with antibodies against GAPDH (1:2000), PIK3R1, p-PIK3R1, Akt, p-Akt, GSK3B, and p-GSK3B (1:1000) overnight at 4 °C. The blot was washed with TBST and incubated with a secondary antibody (1:10,000) at room temperature for 1 h. The protein blots were visualized with ECL reagent after washing again with TBST. Protein expression was quantified by Image J software.

### Statistical analysis

The experimental results were statistically analyzed by SPSS 24.0. The results were expressed as the mean ± standard deviation (SD). Two independent sample t test was applied when the data satisfied a normal distribution. A non-parametric rank-sum test was used for non-normally distributed data. *P* < 0.05 was considered statistically significant.

## Results

### Active components of ACP

**Fig. 2:**
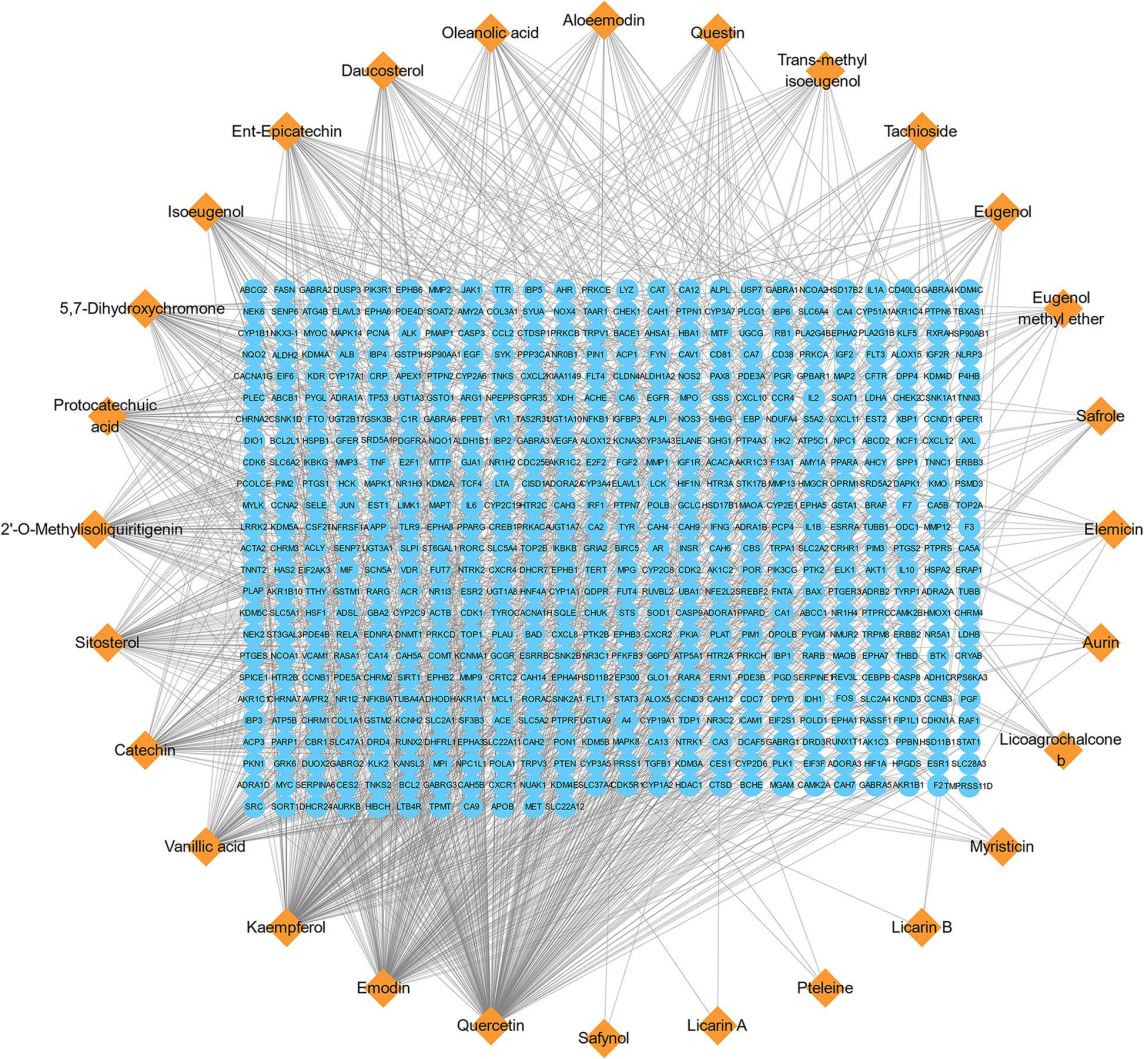
ACP components-target genes network. The networks were generated using Cytoscape 3.7.2 software. Orange diamond nodes represent the compounds of ACP. Blue round nodes represent the target genes of ACP

Eleven ACP components were acquired from the TCMSP database, 31 components were obtained from the HERB database, and 12 components were obtained from NPASS. After deletion of the repeated components, 30 effective compounds were obtained (Table 1). The targets were collected and predicted in the TCMSP, STITCH, SwissTargetPrediction, and SEA databases, and 587 targets were acquired. The components that involve more than 50 target genes include quercetin, emodin, kaempferol, vanillic acid, catechin, sitosterol, 2′-O-Methylisoliquiritigenin, protocatechuic acid, 5,7-dihydroxychromone, and isoeugenol (Table 1, Fig. 2).

**Table 1:** Active ingredients and ADME parameters of ACP

Active compounds	CAS	Molecular Formula	Molecular weight	DL/DW	Gene Number
Quercetin	117–39-5	C_15_H_10_O_7_	302.25	0.28	278
Emodin	518–82-1	C_15_H_10_O_5_	270.25	0.24	189
Kaempferol	520–18-3	C_15_H_10_O_6_	286.25	0.24	183
Vanillic acid	121–34-6	C_8_H_8_O_4_	168.16	0.696	86
Catechin	154–23-4	C_15_H_14_O_6_	290.29	0.24	78
Sitosterol	83–46-5	C_29_H_50_O	414.79	0.75	74
2'-O-Methylisoliquiritigenin	51,828–10-5	C_16_H_14_O_4_	270.3	0.17	70
Protocatechuic acid	99–50-3	C_7_H_6_O_4_	154.13	0.527	68
5,7-Dihydroxychromone	31,721–94-5	C_9_H_6_O_4_	178.15	0.619	57
Isoeugenol	97–54-1	C_10_H_12_O_2_	164.22	0.727	56
Ent-Epicatechin	35,323–91-2	C_15_H_14_O_6_	290.29	0.24	49
Daucosterol	474–58-8	C_35_H_60_O_6_	576.95	0.62	43
Oleanolic acid	508–02-1	C_30_H_48_O_3_	456.78	0.76	35
Questin	3774–64-9	C_16_H_12_O_5_	284.28	0.27	28
Aloeemodin	481–72-1	C_15_H_10_O_5_	270.25	0.24	28
Trans-methyl isoeugenol	6379–72-2	C_11_H_14_O_2_	178.25	0.708	27
Tachioside	109,194–60-7	C_13_H_18_O_8_	302.31	0.19	23
Eugenol	97–53-0	C_10_H_12_O_2_	164.22	0.696	21
Eugenol methyl ether	93–15-2	C_11_H_14_O_2_	178.25	0.66	16
Safrole	94–59-7	C_10_H_10_O_2_	162.2	0.622	13
Elemicin	487–11-6	C_12_H_16_O_3_	208.28	0.696	12
Aurin	603–45-2	C_19_H_14_O_3_	290.33	0.2	10
Licoagrochalcone b	325,144–67-0	C_21_H_20_O_4_	336.4	0.67	9
Myristicin	607–91-0	C_11_H_12_O_3_	192.23	0.689	7
Pteleine	2221–41-2	C_13_H_11_NO_3_	229.25	0.678	3
Licarin b	51,020–87-2	C_20_H_20_O_4_	324.4	0.831	3
Licarin A	2680–81-1	C_20_H_22_O_4_	326.4	0.899	2
Safynol	27,978–14-9	C_13_H_12_O_2_	200.25	0.646	2
Triolein	122–32-7	C_57_H_104_O_6_	885.61	0.13	0
Piperitol	491–04-3	C_10_H_18_O	154.25	0.576	0

### Functional annotation of ACP targets

**Fig. 3:**
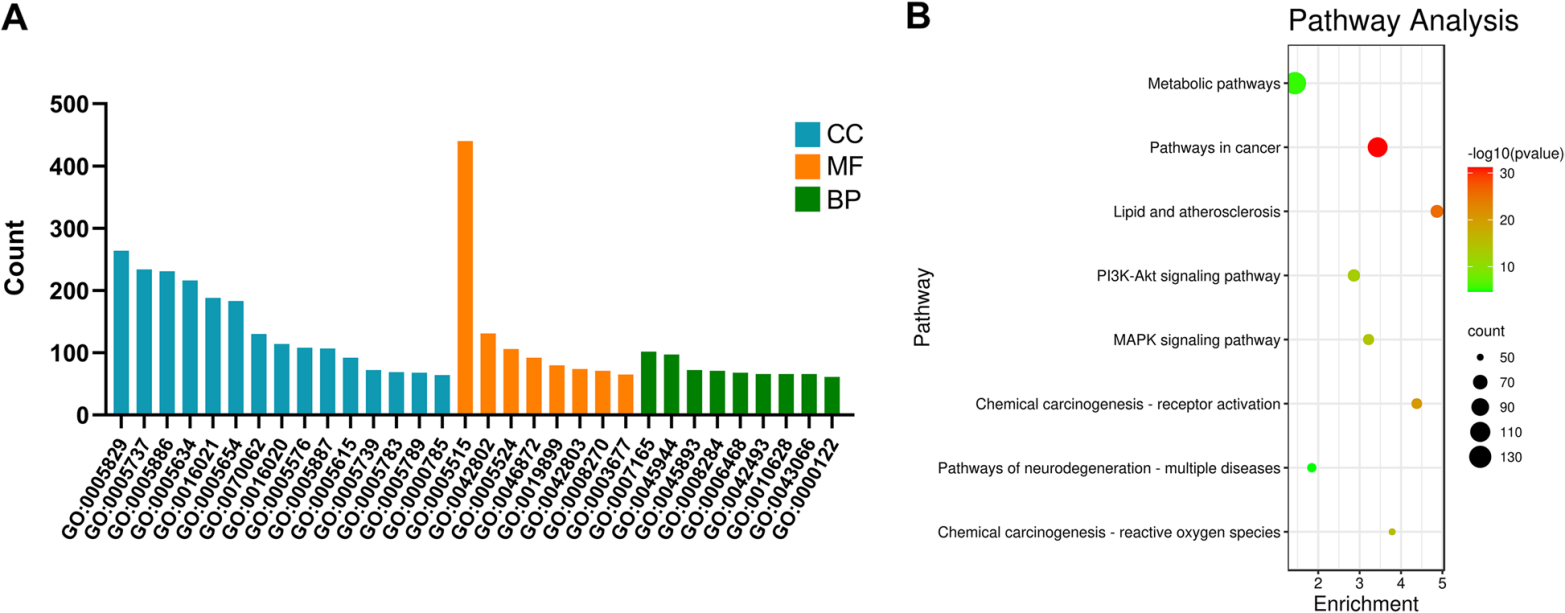
GO functional and KEGG pathway enrichment of ACP target genes (Count numbers ≥ 50). **A** GO enrichment of ACP targets. GO:0005829, cytosol; GO:0005737, cytoplasm; GO:0005886, plasma membrane; GO:0005634, nucleus; GO:0016021, integral component of membrane; GO:0005654, nucleoplasm; GO:0070062, extracellular exosome; GO:0016020, membrane; GO:0005576, extracellular region; GO:0005887, integral component of plasma membrane; GO:0005615, extracellular space; GO:0005739, mitochondrion; GO:0005783, endoplasmic reticulum; GO:0005789, endoplasmic reticulum membrane; GO:0000785, chromatin; GO:0005515, protein binding; GO:0042802, identical protein binding; GO:0005524, ATP binding; GO:0046872, metal ion binding; GO:0019899, enzyme binding; GO:0042803, protein homodimerization activity; GO:0008270, zinc ion binding; GO:0003677, DNA binding; GO:0007165, signal transduction; GO:0045944, positive regulation of transcription from RNA polymerase II promoter; GO:0045893, positive regulation of transcription, DNA-templated; GO:0008284, positive regulation of cell proliferation; GO:0006468, protein phosphorylation; GO:0042493, response to drug; GO:0010628, positive regulation of gene expression; GO:0043066, negative regulation of apoptotic process; GO:0000122, negative regulation of transcription from RNA polymerase II promoter. **B** KEGG pathway enrichment analysis of ACP targets. The dots represent the number of genes, and the shades of color represent the logarithm of the base 10 *P* value

To explore the molecular functions involved in ACP targets, we performed GO functional enrichment analysis using DAVID 6.8. The results showed that these targets were mainly observed in the cytoplasm, plasma membrane, nucleus, cell membrane, mitochondria, endoplasmic reticulum, and other regions of cells, and their molecular functions involved binding to proteins, ATP, enzymes, zinc ions, and sequence-specific DNA. They also participated in biological processes such as signal transduction, positive regulation of transcription from RNA polymerase II promoter, positive regulation of transcription, DNA-template, positive regulation of cell proliferation, protein phosphorylation, response to drug, positive regulation of gene expression, negative regulation of apoptotic process, negative regulation of transcription from RNA polymerase II promoter, apoptotic process, response to xenobiotic stimulus, and inflammatory response (Fig. 3A).

### Pathway enrichment of ACP targets

The results of KEGG pathway enrichment showed that 185 pathways were affected by active components of ACP (*P* < 0.05), and the top 8 pathways (gene numbers ≥ 50) included metabolic pathways, pathways in cancer, lipid and atherosclerosis, PI3K-Akt signaling pathway, MAPK signaling pathway, chemical carcinogenesis-receptor activation, pathways of neurodegeneration-multiple diseases, and chemical carcinogenesis-reactive oxygen species (Fig. 3B). According to the aforementioned findings, ACP might be useful in the treatment of cancer, cardiovascular diseases, and neurological diseases.

### ACP component-colon cancer target gene network

**Fig. 4:**
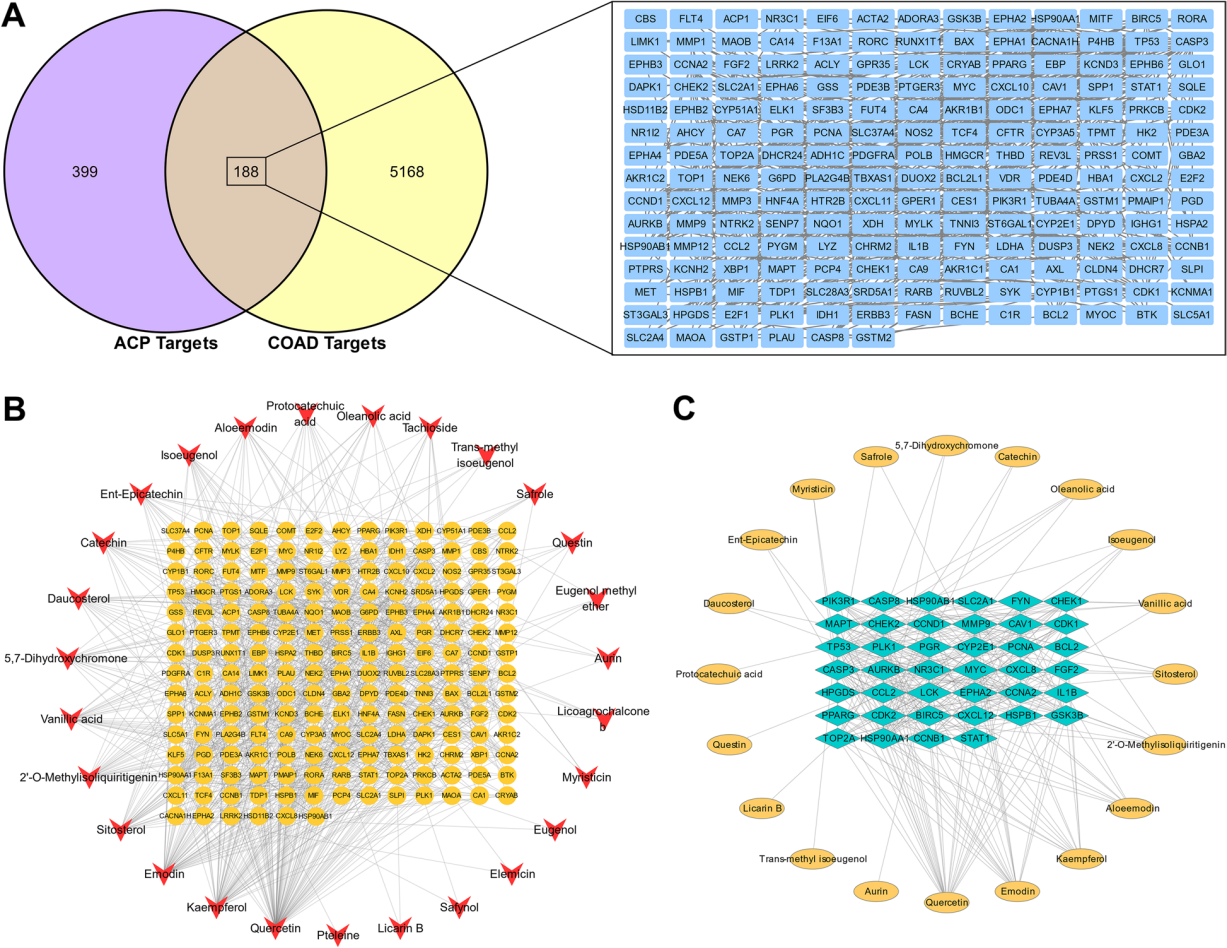
Overlapped targets of ACP and colon cancer. **A** Venn analysis. **B** ACP compound-shared target network. Red V nodes represent the compounds in ACP. Orange round nodes represent shared targets of ACP. Lines indicate interconnections. **C** The key targets of ACP-colon cancer

The GEPIA2 online tool screened 5,356 differentially expressed colon cancer genes, including 2,682 upregulated and 2,674 downregulated genes. The Venn online tool analysis obtained 188 overlapped target genes for ACP-colon cancer (Fig. 4A). PPI was analyzed in the STRING database, in which 158 target genes showed high interactions (confidence score ≥ 0.7). A compound-overlapped target network consisting of 158 nodes and 531 edges was built using Cytoscape 3.7.2 (Fig. 4B). The topological analysis of this network yielded 40 key targets and 199 interactions, all of which exhibited high interaction characteristics (confidence score ≥ 0.7, Fig. 4C, Table 2). Table 3 lists the key targets associated with the main active ingredients of ACP. Quercetin, emodin, kaempferol, and aleemodin had the highest number of key targets of 26, 17, 12, and 8, respectively. Therefore, the four components above might be the key components of ACP for the treatment of colon cancer.

**Table 2:** Forty key targets of ACP acting on colon cancer and related parameters

Gene	Uniprot ID	Degree centrality	Betweenness centrality	Closeness centrality
TP53	P04637	48	0.333	0.514
HSP90AA1	P07900	38	0.261	0.490
MYC	P01106	27	0.086	0.447
CASP3	P42574	25	0.053	0.433
CDK1	P06493	21	0.015	0.412
PIK3R1	P27986	21	0.037	0.387
CCND1	P24385	20	0.026	0.420
FYN	P06241	19	0.041	0.377
HSP90AB1	P08238	18	0.025	0.412
CCNA2	P20248	17	0.006	0.375
MMP9	P14780	17	0.078	0.395
HPGDS	O60760	16	0.140	0.436
CCNB1	P14635	16	0.003	0.373
CHEK1	O14757	16	0.007	0.393
STAT1	P42224	16	0.031	0.416
LCK	P06239	15	0.030	0.372
IL1B	P01584	15	0.017	0.370
FGF2	P09038	15	0.053	0.391
PLK1	P53350	14	0.007	0.398
BIRC5	Q9NQS7	14	0.005	0.371
PCNA	P12004	14	0.009	0.358
CDK2	P24941	14	0.006	0.399
AURKB	Q96GD4	13	0.030	0.391
GSK3B	P49841	13	0.007	0.412
CAV1	Q03135	13	0.029	0.377
TOP2A	P11388	12	0.006	0.356
CXCL12	P48061	12	0.013	0.353
CYP2E1	P05181	11	0.087	0.372
MAPT	P10636	11	0.018	0.397
CCL2	P13500	11	0.005	0.328
CXCL8	P10145	11	0.004	0.336
EPHA2	P29317	10	0.035	0.372
PPARG	P37231	10	0.028	0.414
SLC2A1	P11166	9	0.072	0.380
NR3C1	P04150	9	0.018	0.393
CASP8	Q14790	8	0.004	0.379
BCL2	P10415	8	0.013	0.357
HSPB1	P04792	8	0.004	0.385
CHEK2	O96017	8	0.005	0.349
PGR	P06401	8	0.005	0.395

**Table 3:** Key target genes contained in ACP compounds

Compound	Numbers of key targets	Targets
Quercetin	26	AURKB、CCNB1、TP53、PLK1、BIRC5、HSP90AA1、CDK1、TOP2A、PIK3R1、CASP8、CASP3、BCL2、CCND1、CDK2、GSK3B、MYC、CAV1、HSPB1、IL1B、STAT1、MMP9、MAPT、PPARG、CCL2、CXCL8、CHEK2
Emodin	17	CYP2E1、AURKB、TP53、PLK1、HSP90AB1、HSP90AA1、TOP2A、CASP3、BCL2、CDK2、GSK3B、MYC、LCK、SLC2A1、IL1B、MMP9、PPARG
Kaempferol	12	HSP90AA1、CDK1、TOP2A、CASP3、BCL2、GSK3B、SLC2A1、STAT1、MMP9、MAPT、PPARG、PGR
Aloeemodin	8	CCNB1、TP53、PCNA、HSP90AA1、CASP8、CASP3、MYC、IL1B
2'-O-Methylisoliquiritigenin	7	CCNA2、CHEK1、GSK3B、MMP9、MAPT、PPARG、CXCL12
Sitosterol	6	EPHA2、CASP8、CASP3、BCL2、PGR、FGF2
Vanillic acid	5	CHEK1、FYN、MAPT、CXCL12、CHEK2
Isoeugenol	4	CHEK1、TOP2A、MAPT、CXCL12
Oleanolic acid	4	TP53、HSP90AA1、CASP8、CASP3
Catechin	2	HSP90AA1、TOP2A
5,7-Dihydroxychromone	2	HSP90AA1、TOP2A
Safrole	2	CYP2E1、CASP3
Myristicin	2	HPGDS、CASP3
Ent-Epicatechin	2	CASP3、CCL2
Daucosterol	2	EPHA2、PGR
Protocatechuic acid	1	FYN
Questin	1	CYP2E1
Licarin B	1	TOP2A
Trans-methyl isoeugenol	1	MAPT
Aurin	1	NR3C1

### GO enrichment analysis of ACP key targets

**Fig. 5:**
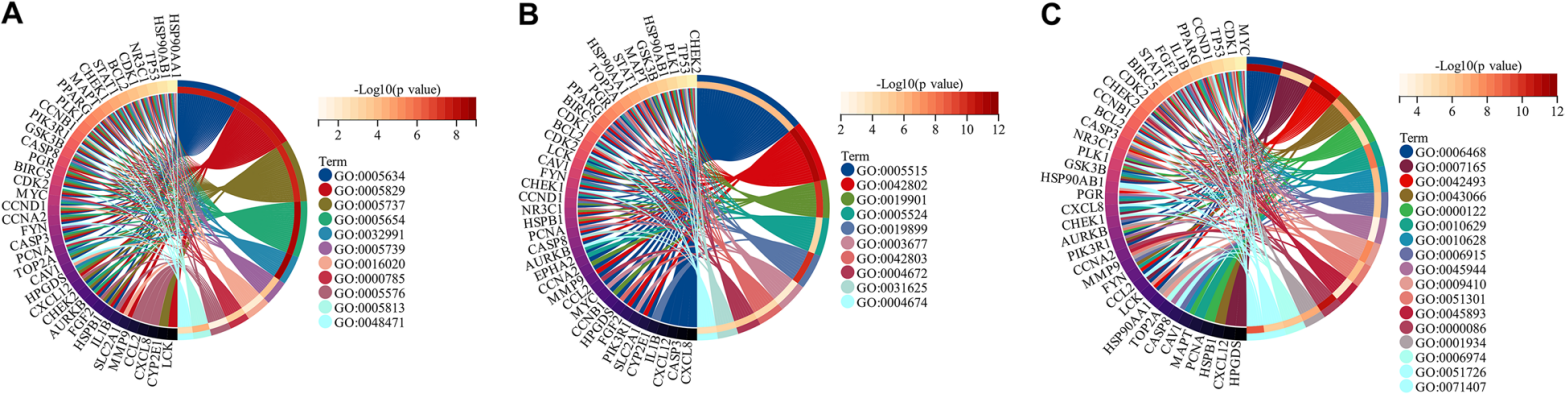
GO functional enrichment of the ACP-colon key targets. **A** Cellular component. GO:0005634, nucleus; GO:0005829, cytosol; GO:0005737, cytoplasm; GO:0005654, nucleoplasm; GO:0032991, macromolecular complex; GO:0005739, mitochondrion; GO:0016020, membrane; GO:0000785, chromatin; GO:0005576, extracellular region; GO:0005813, centrosome; GO:0048471, perinuclear region of cytoplasm. **B** Molecular function. GO:0005515, protein binding; GO:0042802, identical protein binding; GO:0019901, protein kinase binding; GO:0005524, ATP binding; GO:0019899, enzyme binding; GO:0003677, DNA binding; GO:0042803, protein homodimerization activity; GO:0004672, protein kinase activity; GO:0031625, ubiquitin protein ligase binding; GO:0004674, protein serine/threonine kinase activity. **(C)** Biological process. GO:0006468, protein phosphorylation; GO:0007165, signal transduction; GO:0042493, response to drug; GO:0043066, negative regulation of apoptotic process; GO:0000122, negative regulation of transcription from RNA polymerase II promoter; GO:0010629, negative regulation of gene expression; GO:0010628, positive regulation of gene expression; GO:0006915, apoptotic process; GO:0045944, positive regulation of transcription from RNA polymerase II promoter; GO:0009410, response to xenobiotic stimulus; GO:0051301, cell division; GO:0045893, positive regulation of transcription, DNA-templated; GO:0000086, G2/M transition of mitotic cell cycle; GO:0001934, positive regulation of protein phosphorylation; GO:0006974, cellular response to DNA damage stimulus; GO:0051726, regulation of cell cycle; GO:0071407, cellular response to organic cyclic compound

We carried out GO functional enrichment analysis to investigate the role of 40 important ACP target genes in colon cancer in more detail. The results showed that the key target genes of ACP mainly existed in the nucleus, nucleoplasm, cytoplasm, and mitochondria (Fig. 5A). They could bind to molecules such as protein, ATP, double-stranded DNA, and transcription factors, which are associated with the activity of protein kinase and tyrosine/serine/threonine kinase (Fig. 5B). Furthermore, they participated in various bioprocesses such as protein phosphorylation, signal transduction, response to drug, negative regulation of apoptotic process, negative regulation of transcription from RNA polymerase II promoter, negative regulation of gene expression, positive regulation of gene expression, apoptotic process, positive regulation of transcription from RNA polymerase II promoter, response to xenobiotic stimulus, cell division, positive regulation of transcription, DNA-template, G2/M transition of mitotic cell cycle, positive regulation of protein phosphorylation, cellular response to DNA damage stimulus, regulation of cell cycle, and cellular response to organic cyclic compound (Fig. 5C).

### Pathway enrichment analysis of ACP key targets

**Fig. 6:**
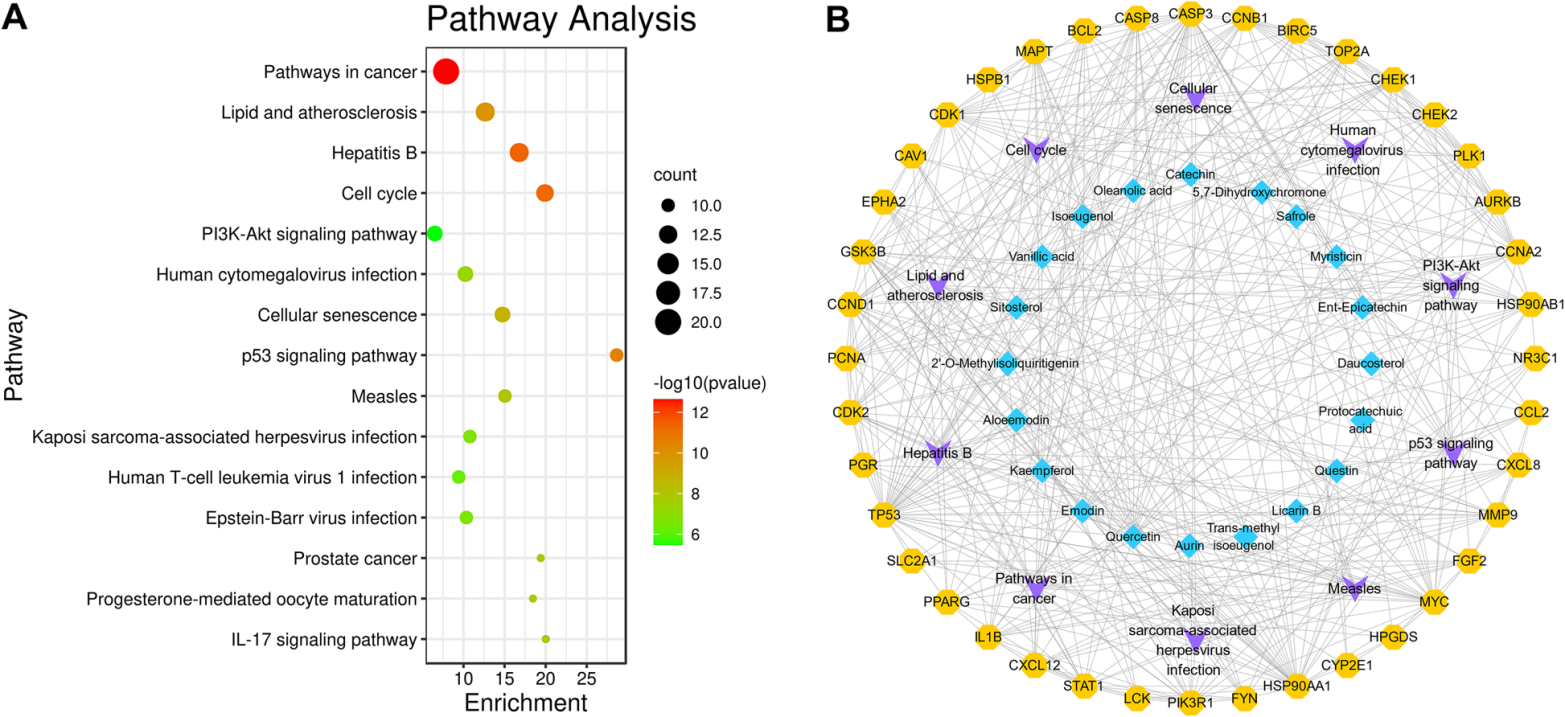
Pathway enrichment of the ACP-colon key targets. **A** KEGG pathway enrichment analysis. **B** Compound–key target–pathway network. Blue diamond nodes represent the compounds in ACP. Violet V nodes represent the pathway. Orange octagon nodes represent the key targets of ACP

**Fig. 7:**
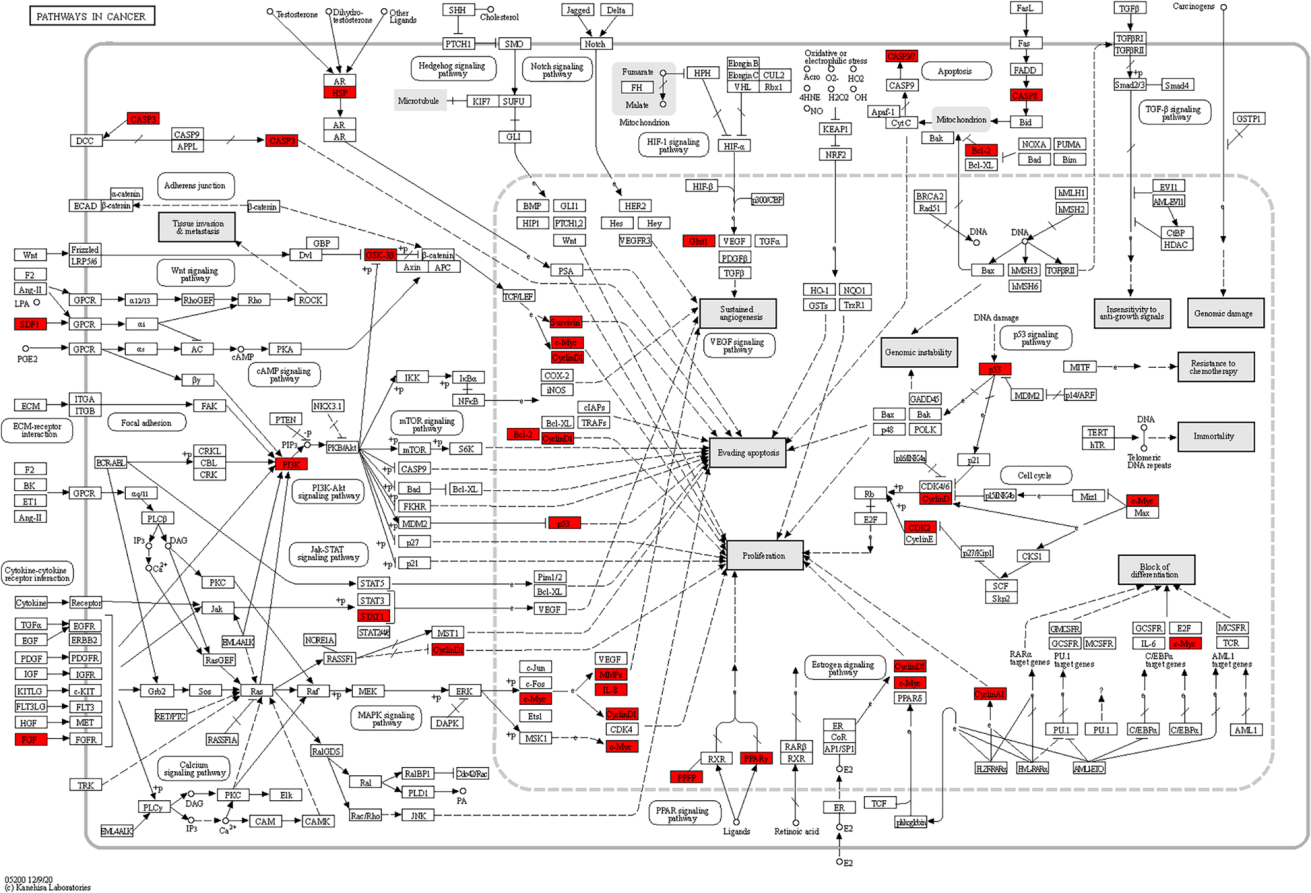
Distribution of the ACP-colon key targets in pathways in cancer. The red rectangles stand for the key targets (KEGG Copyright Permission 232293)

Next, we used KEGG pathway enrichment analysis to investigate the main signaling pathways associated with the target genes. The findings indicated that 92 pathways were affected by the key targets of ACP (*P* < 0.05), mainly including pathways in cancer, hepatitis B, lipid and atherosclerosis, cell cycle, cellular senescence, human cytomegalovirus infection, PI3K-Akt signaling pathway, p53 signaling pathway, measles, kaposi sarcoma-associated herpesvirus infection, Epstein-Barr virus infection, and human T-cell leukemia virus 1 infection (Fig. 6A). The above results suggested that crucial pathways for ACP to treat colon cancer include pathways in cancer, cell cycle, cellular senescence, PI3K-Akt, and p53 signaling pathways. Furthermore, a number of targets are enriched in the viral infection pathways linked to the development of cancer, indicating that ACP may have anticancer effects through mechanisms including antiviral infection. The compound-key target-pathway network is displayed in Fig. 6B. The key target distribution in pathways in cancer is mapped in Fig. 7.

### Molecular docking

**Fig. 8:**
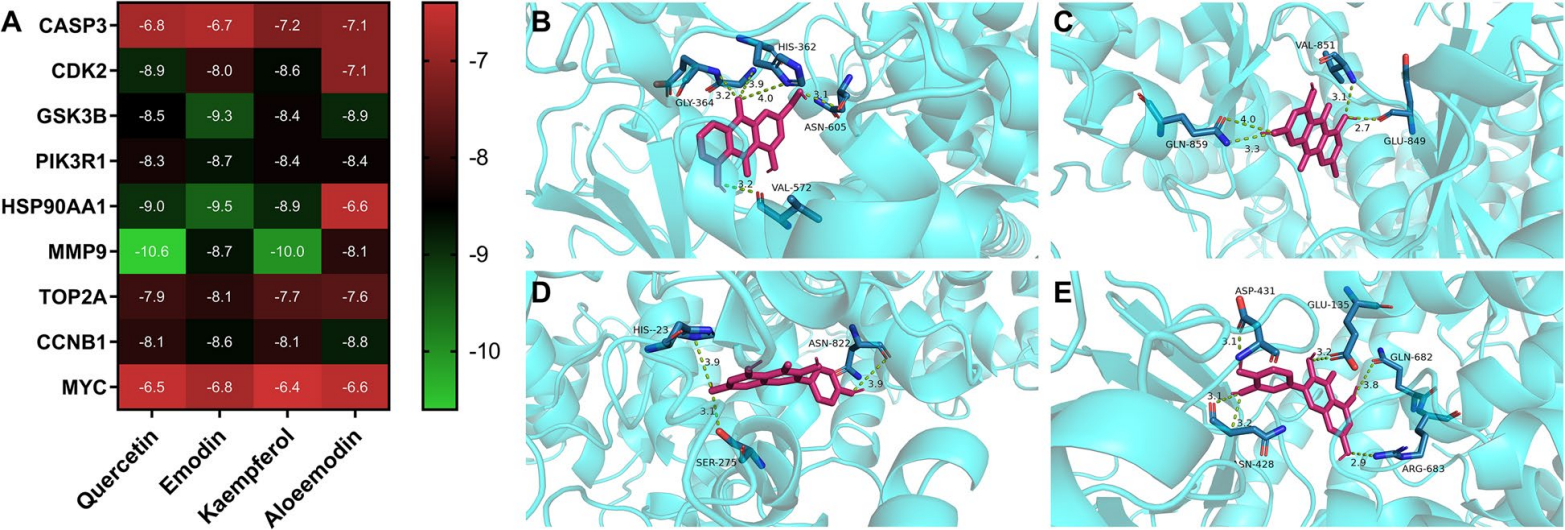
Molecular docking. **A** Molecular docking was carried out using a heatmap. The numbers in the figure represent the affinity score. **B** Aloeemodin binding with PIK3R1. **C** Emodin binding with PIK3R1. **D** Kaempferol binding with PIK3R1. **E** Quercetin binding with PIK3R1

The key compounds in ACP, quercetin, emodin, kaempferol, and aloeemodin, were selected for molecular docking analysis with the corresponding key targets. The lower the binding energy of the ligand to the receptor, the more stable the conformation of the two. After screening based on the criterion of affinity score ≤ -5 kJ/mol, the results are shown in Fig. 8A. Quercetin, emodin, kaempferol, and aloeemodin can bind to multiple targets stably. The binding sites of compounds and key targets were visualized using the PLIP website and PyMOL v.3.8 software, and the binding of quercetin, emodin, kaempferol, and aloeemodin to PIK3R1 is shown in Fig. 8B. Five hydrogen bonds were established between aloeemodin and PIK3R1 via GLY-364, HIS-362, ASN-605, and VAL-572. Four hydrogen bonds were established between emodin and PIK3R1 via GLN-859, VAL-851, and GLU-849. Three hydrogen bonds are formed between kaempferol and PIK3R1 via SER-275, ASN-822, and HIS-23. Quercetin forms 6 hydrogen bonds with PIK3R1 via ASN-428, ASP-431, GLU-135, GLN-682, and ARG-683.

### Anticancer effects of ACP on CT26 cells

**Fig. 9:**
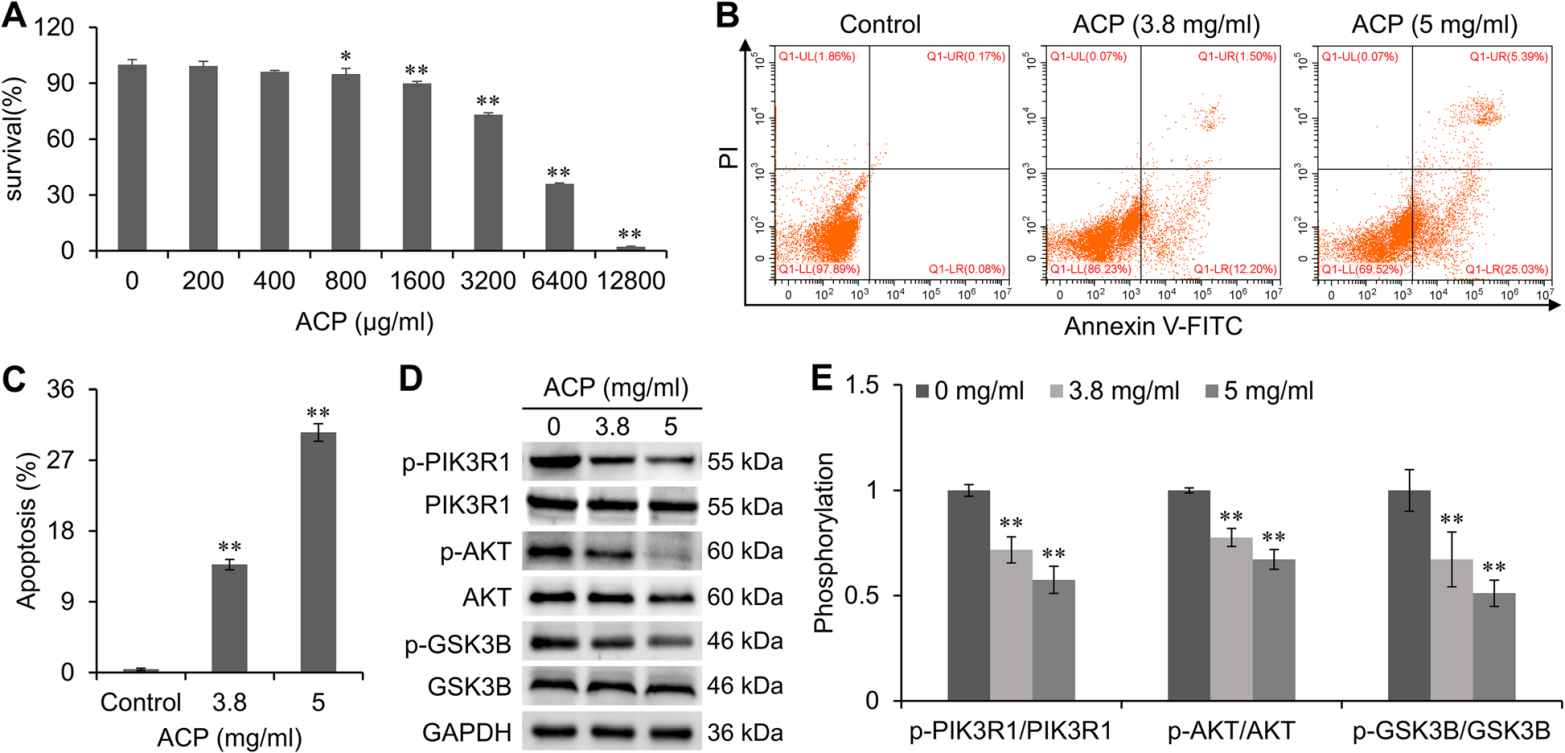
Anticancer effects of ACP against CT26 cells. **A** Cell proliferation was detected using CCK-8 assay. **B**, **C** Apoptosis was identified by flow cytometry. **D**, **E** proteins expression and phosphorylation were evaluated by Western blot. Compared with control group, *P < 0.05, **P < 0.01

In order to clarify the effect of ACP on colon cancer CT26 cells, we chose the CCK-8 assay to detect the cell viability of CT26 cells after treatment with different concentrations of ACP. The results showed that 1600–12800 μg/ml ACP significantly inhibited the proliferation of CT26 cells (*P* < 0.01) in a dose-dependent manner (Fig. 9A). A low concentration of 3.8 mg/ml and a high concentration of 5 mg/ml were selected for subsequent experiments. Annexin V-FITC/PI double staining and flow cytometry analysis showed that 3.8 mg/ml and 5 mg/ml of ACP induced apoptosis in CT26 cells (*P* < 0.01), as shown in Fig. 9B and C. The expression and phosphorylation of PIK3R1 and its downstream proteins were further detected by Western blot. The results showed that ACP did not affect the expression of PIK3R1, AKT, and GSK3B but inhibited the phosphorylation of PIK3R1, AKT, and GSK3B (Fig. 9D and E). The above results demonstrated that the PI3K/Akt signaling pathway may be an important pathway involved in ACP-induced apoptosis.

## Discussion

Recent years have seen a dramatic shift in people's eating patterns and lifestyles due to the ongoing development of material living conditions. High-fat and low-dietary fiber diets, a lack of physical exercise, and other factors have contributed to the increasing incidence of CRC year by year, creating a greater health and economic burden. Existing treatments are effective in prolonging the survival time of patients with advanced CRC, but the 5-year survival rate of metastatic patients is still low, and all of these treatments are accompanied by varying degrees of side effects and complications [31]. TCM has unique advantages in the treatment of CRC by inhibiting tumor cell proliferation and inducing apoptosis. TCM alone or in combination with chemotherapy drugs in the synergistic treatment of advanced CRC can prolong the survival time of patients while ensuring their quality of life [32]. ACP is a commonly used anti-tumor TCM in clinical practice and is often combined with other herbs to form an herbal formula for CRC treatment. We have developed Tenglong Buzhong decoction (TBD; Chinese Patent No. ZL200910197565.2), which consists of *A. chinensis* Planch (Tengligen), *Solanum nigrum* L. (Longkui), *Duchesnea indica* (Andr.) Focke (Shemei), *Scutellaria barbata* D. Don (Banzhilian), and *Viscum coloratum* (Kom.) Nakai (Hujisheng). TBD can inhibit the growth of CRC, reduce the adverse effects of chemotherapy, relieve symptoms, and improve the immune function and quality of life in patients with CRC [33–35]. Despite proven efficacy, the pharmacologic mechanism of ACP in CRC treatment is unclear.

In the present study, the key components and potential targets of ACP for the treatment of colon cancer were studied via network pharmacology and molecular docking to provide a feasible basis for the study and application of ACP. The results show that quercetin, emodin, kaempferol, and aloeemodin are effective, active components in ACP that exerts anti-colon cancer effect. Quercetin is a natural flavonoid that has powerful anti-tumor and antioxidant effects [36]. Numerous investigations have indicated that quercetin can exert an anti-CRC proliferation, invasion, and pro-apoptosis effects by regulating the signaling pathways of PI3K-Akt, Wnt/β-catenin, the p53 signaling pathway, and the MAPK cascade [37]. In addition, studies have shown that the combination of quercetin and oxaliplatin can synergically inhibit glutathione reductase activity in human colon cancer HCT116 cells, reduce intracellular glutathione levels, increase ROS production, and reduce cell viability. In the HCT116 xenograft mouse model, tumor growth was significantly inhibited. These results suggested that quercetin can enhance the anticancer effect of oxaliplatin by depleting intracellular glutathione [38]. Emodin is a natural anthraquinone derivative with anti-tumor, antioxidant, hypoglycemic, and anti-inflammatory properties. Zhang et al. [39] investigated the effects of emodin on colonic inflammation and tumorigenesis by constructing an azoxymethane/dextran sodium sulfate (AOM/DSS) colitis-associated intestinal cancer mouse model. The results showed that emodin attenuated the inflammatory response in the tumor microenvironment, reduced inflammatory cells CD11b and F4/80 recruitment, and decreased the cytokines tumor necrosis factors alpha (TNFα), interleukin-1 alpha/beta (IL1α/β), IL6, and the pro-inflammatory enzymes cyclooxygenase-2 (COX2) and nitric oxide synthase 2 (NOS2) in the tumor microenvironment. In vitro experiments showed that emodin reduced the viability of SW620 and HCT116 cells, as well as inhibited their adherence, migration, and invasion abilities. Kaempferol, a yellow tetrahydroxy flavonoid, is now thought to be a possible natural anticancer agent that combats cancer in a number of ways, including liver, stomach, breast, and colon malignancies [40]. Wu et al. [41] found that kaempferol could directly inhibit the expression of pyruvate kinase M2 isoform (PKM2) or indirectly block the alternative splicing factors of PKM mRNA in HCT-8R cells, which in turn reduced glucose uptake and lactate production in drug-resistant CRC cells. This effectively reversed 5-fluorouracil (5-FU) resistance. Aloeemodin is a small molecule of hydroxy-anthraquinone found in natural plants that has a wide range of anti-tumor effects. Jiang et al. [42] discovered that following aloeemodin therapy, HCT116 cell cytoplasmic protein expressions of Bax and cytochrome C increased, whereas the mitochondrial protein expressions of Bcl-2 decreased. It is suggested that aloeemodin may play an anti-apoptotic role by activating the mitochondrial pathway.

In addition to the above four key ingredients, other ingredients also affect the treatment of colon cancer. Sitosterol can effectively block the Wnt/β-catenin pathway by down-regulating the expression of the lymphoid enhancer-binding factor 1 (LEF-1) gene and its downstream targets, c-myc, survivin, and CCND1, and exerting anti-HCT116 cell proliferation and invasion effects [43]. Oleanolic acid induces both apoptosis and pro-survival mitophagy via the p38/forkhead box O3a (FOXO3a)/Sirt6 signaling in colon cancer cells, and also increases the chemosensitivity of colon cancer cells to 5-Fu [44]. Furthermore, GO functional and KEGG pathway enrichment analyses revealed that the targets of ACP existed in multiple cellular areas and involved diversified molecular functions. They were also correlated with pathways such as metabolic pathways, pathways in cancer, lipid and atherosclerosis, PI3K-Akt signaling pathway, MAPK signaling pathway, chemical carcinogenesis-receptor activation, pathways of neurodegeneration-multiple diseases, and chemical carcinogenesis-reactive oxygen species, suggesting that ACP has a pharmacological role not only in oncological diseases but also in cardiovascular, cerebrovascular, and neurodegenerative-related diseases.

In the present study, 40 key target genes of ACP for colon cancer treatment were identified. Caspase-3 (CASP3) is an apoptosis-related protein; Cyclin-dependent kinase 2 (CDK2) and cell cycle-related proteins cyclin B1 (CCNB1) are cell cycle regulatory proteins. Phosphoinositide-3-kinase regulatory subunit 1 (PIK3R1) is an important protein in the PI3K/Akt signaling pathway, and it is related to cell proliferation and apoptosis. Matrix metallopeptidase 9 (MMP9) is a matrix metalloproteinase that is closely related to metastasis. Glycogen synthase kinase 3 beta (GSK3B) is a multifunctional serine/threonine kinase that is involved in regulating cellular activities such as cell proliferation, differentiation, apoptosis, metabolism, and stem cell renewal, and its polymorphisms are associated with the development of CRC [45]. Topoisomerase II alpha (TOP2A) is highly expressed in colorectal cancer and is involved in the regulation of cell proliferation, apoptosis, and invasion [46, 47]. Molecular docking results show that the key components of ACP, quercetin, emodin, kaempferol, and aloeemodin have good binding activities with the corresponding key target proteins.

The key targets of ACP, according to GO and pathway enrichment studies, may bind to proteins and double-stranded DNA, control the actions of protein kinases like serine, threonine, and histone, and participate in protein dimerization and other activities, thus influencing the pathways in cancer, hepatitis B, lipid, and atherosclerosis, cell cycle, cellular senescence, human cytomegalovirus infection, PI3K-Akt signaling pathway, p53 signaling pathway, measles, kaposi sarcoma-associated herpesvirus infection, Epstein-Barr virus infection, human T-cell leukemia virus 1 infection, IL-17 signaling pathway, prostate cancer, and progesterone-mediated oocyte maturation. They also participate in bioprocesses such as drug response, signal transduction, angiogenesis, cell migration, apoptosis, and cell cycle. Pathways in cancer are a collection of multiple pathways involving Wnt, PI3K-Akt, MAPK, and other tumor-related signaling pathways. The Wnt/β-catenin signaling pathway is a recognized driver of CRC. As a functional effector molecule of signaling, the modification and degradation of β-catenin is a key factor in the development of CRC and can participate in proliferation, stemness, apoptosis, EMT, invasion, and metastasis of CRC cells [48]. The PI3K/Akt signaling pathway is an important signaling pathway for the occurrence and development of various cancers, which can participate in the regulation of tumor cell survival, metastasis, and metabolism [49]. PI3K is an important member of the phospholipid kinase family. Akt, also known as protein kinase B (PKB), is an important signaling molecule downstream of PI3K, and its different isoforms also play important roles in cancer [50]. Abnormal activation of the PI3K/Akt pathway is often predictive of the development of CRC and drug resistance [51]. Therefore, it is crucial to understand the role of this pathway in CRC. The p53 protein is a classical tumor suppressor gene with critical functions in maintaining genomic integrity and is involved in the regulation of the cell cycle, DNA repair, senescence, and apoptosis [52]. Three significant subfamilies of MAPK are involved in the pathophysiology of colorectal cancer (CRC): ERK, c-Jun N-terminal kinase or stress-activated protein kinases (JNK or SAPK), and MAPK14. When triggered by varied stimuli, the MAPK pathway can control the growth, differentiation, apoptosis, migration, and invasion of tumor cells [53].

Finally, based on the combination of KEGG pathway enrichment analysis and molecular docking results, we selected the PI3K-Akt signaling pathway as the target and observed the anticancer effects of ACP in vitro. The results showed that ACP inhibited the proliferation of CT26 cells in a dose-dependent manner, induced apoptosis, and inhibited the phosphorylation of PIK3R1, AKT, and GSK3B. PIK3R1 is the regulatory subunit of PI3K that could activate AKT through phosphorylation and then inhibit GSK3B through phosphorylation. GSK3B inhibits the expression of its downstream β-catenin and its target genes survivin, c-Myc, and cyclin D1 [54]. Survivin is a member of the inhibitor-of-apoptosis protein (IAP) family and resists caspase-dependent apoptosis [55]. C-Myc is an important regulator in cellular programs. Aberrations in the Myc gene or up-regulation of its expression through related pathways can occur in most cancers [56]. Cyclin D1 is a cell cycle regulatory protein, and its abnormal isoforms can participate in important processes such as tumor cell cycle, invasion, and metastasis [57]. Taken together, ACP could inhibit the phosphorylation of PIK3R1, AKT and GSK3B, suggesting that the effect of ACP is related to the regulation of PI3K/AKT/GSK3B signal transduction.

However, our study has the following limitations: The relevant information included in the existing drug and target gene databases may be incomplete, which reduces the credibility of the prediction results; the role of ACP in the regulation of GSK3B downstream signaling molecules needs to be further investigated; and the present study only addressed the apoptosis-inducing effect of ACP in colon cancer cells and did not delve deeper into other roles of ACP on CRC such as invasion and metastasis. Nevertheless, our findings provide important preliminary information on the role of ACP in anti-CRC therapy, suggesting that ACP may be a potentially effective drug for the treatment of CRC.

## Conclusion

In summary, the active components of ACP for the treatment of colon cancer include quercetin, emodin, kaempferol, and aloeemodin, and the effective targets include CASP3, CDK2, GSK3B, PIK3R1, HSP90AA1, MMP9, TOP2A, CCNB1, and MYC, with pathways in cancer, cell cycle, cellular senescence, the PI3K-Akt signaling pathway and the p53 signaling pathway, which contribute to multiple bioprocesses, such as drug response, signal transduction, angiogenesis, cell migration, apoptosis, and cell cycle. ACP can also inhibit cell proliferation and induce apoptosis through a mechanism related to the regulation of PI3K/AKT/GSK3B signaling. This study systematically analyzed the active components, target genes, and signaling pathways of ACP in the treatment of colon cancer, thus providing a basis for the application and study of ACP.

## Data Availability

The datasets used and/or analyzed during the current study available from the corresponding author on reasonable request.

## Abbreviations

ACP: *Actinidia chinensis* Planch
PPI: Protein- protein interaction network
GO: Gene ontology
KEGG: Kyoto Encyclopedia of Genes and Genomes
CRC: Colorectal cancer
TCM: Traditional Chinese medicine
ROS: Reactive oxygen species
E2F1: E2F transcription factor 1
TCMSP: Traditional Chinese Medicine Systems Pharmacology Database and Analysis Platform
ETCM: Encyclopedia of Traditional Chinese Medicine
NPASS: Natural Product Activity and Species Source Database
OB: Oral bioavailability
DL: Drug-likeness
DW: Drug-likeness weight
SEA: Similarity Ensemble Approach
TCGA: The Cancer Genome Atlas
GTEx: Genotype-tissue expression
GEPIA2: Gene Expression Profiling Interactive Analysis
DC: Degree centrality
BC: Betweenness centrality
CC: Closeness centrality
PDB: Protein Data Bank
CCK-8: Cell counting kit-8
SD: Standard deviation
AOM/DSS: Azoxymethane/dextran sodium sulfate
TNFα: Tumor necrosis factors alpha
IL1α: Interleukin-1 alpha
COX2: Cyclooxygenase-2
NOS2: Nitric oxide synthase 2
5-FU: 5-Fluorouracil
LEF-1: Lymphoid enhancer-binding factor 1
FOXO3a: Forkhead box O3a
CASP3: Caspase-3
CDK2: Cyclin-dependent kinase 2
CCNB1: Cell cycle-related proteins cyclin B1
PIK3R1: Phosphoinositide-3-kinase regulatory subunit 1
MMP9: Matrix metallopeptidase 9
GSK3B: Glycogen synthase kinase 3 beta
TOP2A: Topoisomerase II alpha
PKB: Protein kinase B
JNK: C-Jun N-terminal kinase
SAPK: Stress-activated protein kinases
IAP: Inhibitor-of-apoptosis protein
